# Expanding the clinical spectrum of pediatric CASPR2 antibody-associated autoimmune encephalitis: a multicenter case series

**DOI:** 10.3389/fimmu.2026.1820277

**Published:** 2026-07-08

**Authors:** Fanni Szumutku, Léna Szabó, Zoltán Liptai, Beáta Rosdy, Gabriella Merő, Márk Merza, Andrea Skobrák, Csaba Zsiborás, Eszter Barabás, Sarolta Dobner

**Affiliations:** 1 Tűzoltó Street Department, Pediatric Center, Semmelweis University, Budapest, Hungary; 2 Heim Pál National Pediatric Institute, Budapest, Hungary; 3 Pediatric Clinic, University of Debrecen, Debrecen, Hungary; 4 Győr-Moson-Sopron County Petz Aladár University Teaching Hospital, Győr, Hungary; 5 Somogy County Kaposi Mór Teaching Hospital, Kaposvár, Hungary; 6 Department of Pediatrics, University of Pécs, Pécs, Hungary; 7 Immunology Laboratory, Department of Laboratory Medicine, Semmelweis University, Budapest, Hungary

**Keywords:** Caspr2, CASPR2 antibody, CASPR2 autoimmune encephalitis, neuroinflammation, pediatric autoimmune encephalitis

## Abstract

**Introduction:**

Contactin-associated protein-like 2 (CASPR2) antibody-associated autoimmune encephalitis is rare in children, and available clinical data are limited. Our aim is to describe the clinical spectrum, diagnostic findings, treatment, and outcomes of all pediatric CASPR2 antibody-associated encephalitis patients reported in Hungary.

**Methods:**

We present a retrospective case series including nine pediatric patients diagnosed with CASPR2 antibody-associated encephalitis across five centers. Clinical, laboratory, imaging, electrophysiological findings, treatment, and follow-up data were collected.

**Results:**

The mean age at onset was 4.1 ± 2.6 years. All patients were serum CASPR2 antibody-positive; two had CSF positivity. The most common symptoms included sleep disorder, irritability, aggressiveness, hypertension, tachycardia, abdominal pain, itching, exanthema, and weight loss. No specific abnormalities were detected on MRI, EEG, or laboratory testing. Immunotherapy led to favorable outcomes in nearly all patients, with one relapse during follow-up.

**Discussion:**

This series expands the clinical spectrum of CASPR2-associated autoimmune encephalitis in children. The presenting symptoms in pediatric patients may differ not only from those observed in adults but also from other forms of autoimmune encephalitis. The diagnostic process is especially complex due to the variability and age-related evolution of clinical manifestations, which can be difficult to recognize in younger children and commonly contribute to diagnostic delay. Early recognition and immunotherapy are associated with favorable prognosis. This case series underscores the importance of recognizing the diverse spectrum of presenting symptoms and highlights the need for raising awareness of the clinical variability. Larger prospective studies are needed to define diagnostic, therapeutic strategies and prognostic factors in the pediatric population.

## 1 Introduction

Contactin-associated protein-like 2 (CASPR2) is a transmembrane protein expressed in neurons of both the central and peripheral nervous systems, and represents one of the primary antigens within the voltage-gated potassium channel complex (1).

CASPR2 antibody-associated autoimmune encephalitis (hereafter referred to as CASPR2 encephalitis) is a rare autoimmune neurological disorder (2). According to a recent systematic review, only 40 pediatric cases have been reported to date, and data on affected children remain limited (2). The clinical spectrum is highly heterogeneous, often leading to delays in diagnosis and treatment (2). CASPR2 encephalitis may present with diverse syndromes and overlapping phenotypes involving the central, peripheral, and autonomic nervous systems in varying combinations (3–5), including limbic encephalitis, Morvan syndrome, peripheral nerve hyperexcitability syndrome, cerebellar syndrome or painful neuropathy (3, 4, 6). In adults, the core clinical features typically include cognitive impairment, seizures, cerebellar dysfunction, peripheral nerve hyperexcitability, insomnia, autonomic dysfunction, neuropathic pain and weight loss (3, 5).

To date, no randomized controlled trials have evaluated the efficacy of treatment strategies for CASPR2 encephalitis. Immunotherapy usually consists of corticosteroids and/or intravenous immunoglobulin (IVIG) or plasma exchange (PEX), whereas second-line immunosuppressive therapies may be required in some cases, similarly to other autoimmune encephalitides (2). Relapse, however, appears to be exceedingly rare in pediatric patients (1).

Current understanding of the disease’s clinical presentation, diagnostic approach, and treatment response remains limited (2, 5). Therefore, continued reporting of the incidence, clinical features, phenotypes, diagnostic strategies, treatment modalities, prognoses and outcomes of affected pediatric patients is essential to improve understanding of this condition (7).

Accordingly, this case series aims to expand current knowledge by presenting the clinical characteristics, diagnostic findings, therapeutic interventions and outcomes of nine pediatric patients with CASPR2 encephalitis.

## 2 Methods

Patient data were retrospectively reviewed between 2017 and 2025 at one of the participating centers. During this period, 81 children with suspected encephalitis were evaluated. The mean age of the patients was 6.38 ± 5.74 years and the female-to-male ratio was 1:1.25. Autoimmune encephalitis panel testing was performed in 36 patients because of suspected autoimmune etiology. The panel yielded positive results in 11 cases, including 4 patients with CASPR2 encephalitis. The study was approved by the Regional and Institutional Scientific Research Ethics Committee of Semmelweis University (SE RKEB 220/2025).

Additional data from 5 patients were collected from other centers, however, comparably detailed clinical data on all pediatric patients with suspected encephalitis were not available. Written informed consent was obtained from the legal guardians of all participants, and written informed consent for publication of the anonymized clinical image of Patient 4 was obtained prior to publication. To the best of the authors’ knowledge, this cohort comprised all pediatric patients diagnosed with CASPR2 encephalitis in Hungary during the aforementioned time interval.

Antibody detection was performed in both serum and cerebrospinal fluid (CSF) using the indirect immunofluorescence method with a diagnostic kit containing biochips with fixed transfected HEK293 cells (IIFT: Autoimmune Encephalitis Mosaic 6, Euroimmun, Lübeck, Germany). The encephalitis panel included testing for antibodies against leucine-rich glioma-inactivated 1 (LGI1), CASPR2, dipeptidyl-peptidase–like protein 6 (DPPX), γ-aminobutyric acid (GABA) B1/B2 receptor, N-methyl-D-aspartate receptor (NMDAR), and α-amino-3-hydroxy-5-methyl-4-isoxazolepropionic acid receptor (AMPAR). All analyses were conducted according to the manufacturer’s instructions. In 7 of 9 patients an indirect immunofluorescence assay using a commercial fixed composite substrate, comprising monkey cerebellar tissue (Inova Nova Lite Monkey Cerebellum/Cerebrum and Mouse Stomach Slides) was also performed (data not shown).

Demographic, clinical, laboratory, imaging, electrophysiologic findings, treatment and follow-up data were collected retrospectively from medical records for patients with CASPR2 encephalitis. Modified Rankin Scale (mRS) score was used to retrospectively assess the disease severity at admission, discharge and last follow-up. Descriptive statistical analyses were performed in Stata 19 BE (StataCorp LLC, College Station, TX, USA). Continuous variables are presented as mean ± standard deviation (SD).

## 3 Results

### 3.1 Patient cohort

Nine patients with serum and/or CSF CASPR2 antibodies were identified (**Table 1**). The female-to-male ratio was 1:1.25, and the mean age at disease onset was 4.1 ± 2.6 years. All patients had normal early development, were fully immunized according to the national vaccination schedule, and had no significant medical history except for Patient 9, who had previously been treated for epilepsy.

**Table 1:** Main clinical characteristics, abnormal laboratory and imaging findings.

Parameter	Patient 1	Patient 2	Patient 3	Patient 4	Patient 5	Patient 6	Patient 7	Patient 8	Patient 9
Sex/Age at onset (years)	F/3	M/6	F/1	F/3	M/1	M/7	M/2	F/2	M/8
Viral prodrome	URTI	No	URTI	gastroenteritis	no	URTI	no	no	no
Latency	3 weeks+6 days	–	1 week	12 weeks +3 days	–	8 weeks	–	–	–
Neurologic examination	somnolence, anisocoria, pyramidal signs, areflexia, ataxia, hypotonia	somnolence, areflexia, hypotonia, proximal muscle weakness	somnolence, pyramidal sign, hypotonia	somnolence	somnolence, ataxia	pyramidal signs	somnolence, mutism, ataxia, hypotonia, muscle weakness in the lower extremities	normal	normal
EEG	severe diffuse, generalized slowing	normal	moderate to severe generalized slowing	normal	normal	moderate to severe generalized slowing	normal	normal	diffuse dysfunction in sleep, generalized slowing
Neuroimaging findings	normal	normal	T2/FLAIR hyperintensities involving focal cortical/subcortical regions	normal	normal	T2/FLAIR hyperintense lesion involving the right parieto-occipital and frontal cortex and subcortical white matter	T2/FLAIR hyperintensity involving the hippocampus and amygdala	normal	normal
Serum WBC	25.3 × 10^9^/L	13.2 × 10^9^/L	16.72 × 10^9^/L	20.27 × 10^9^/L	normal	11.45 × 10^9^/L	18.53 × 10^9^/L	11.9 × 10^9^/L	normal
Other laboratory findings in the serum	hyponatremia	–	elevated renin-angiotensin levels	–	–	–	hyponatremia	–	–
CSF WBC	4/uL	1/uL	3/uL	1/uL	3/uL	3/uL	ND	1/uL	2/uL
CSF protein level (150–450 mg/L)	890 mg/L	194 mg/L	165 mg/L	253 mg/L	290 mg/L	390 mg/L	220 mg/L	187 mg/L	310 mg/L
CASPR2 antibodies serum	1:160	1:320	1:160	1:320	1:5120	1:40	++*	+++*	1:20
CASPR2 antibodies CSF	–	–	–	–	1:10	–	++*	-*	–
LGI antibodies serum	–	1:20	1:40	1:80	1:160	–	+	–	–
LGI antibodies liquor	–	–	–	–	–	–	+	–	–
Diagnostic delay	9.6 weeks	14.6 weeks	5 weeks	11.9 weeks	8.3 weeks	8 weeks	2.1 weeks	8 weeks	1.9 weeks
Treatment	HDMP, IVIG	HDMP, IVIG	HDMP, IVIG relapse: HDMP, AZA	HDMP	HDMP, IVIG	IVIG	HDMP, IVIG	no immunotherapy	HDMP, PEX, AZA
mRS onset/discharge	5/2	4/2	3/0	2/1	2/0	4/0	5/2	2/0	2/1
Follow-up period	7.6 years	1.1 years	1.9 years	0.75 years	4.4 years	1.8 years	6 years	0.4 years	3.8 years

AZA, azathioprine; CBC, complete blood count; F, female; IVIG, intravenous immunoglobulin; HDMP, high-dose methylprednisolone; M, male; mRS, Modified Rankin Scale; ND, no data; PEX, plasma exchange; URTI, upper respiratory tract infection; WBC, white blood cell.

*The examination was performed in an external laboratory, where “+” denotes a titer of 1:100.

### 3.2 Clinical presentation

A prodromal illness was observed in 4/9 patients, most commonly an upper respiratory tract infection (3/4 patients, **Table 1**). The initial neurological symptoms following the prodromal phase are summarized in **Table 2**. Pain was the initial symptom in 6/9 patients; however, its presentation varied considerably in younger children.

**Table 2:** Presenting symptoms.

Patient 1	Patient 2	Patient 3	Patient 4	Patient 5	Patient 6	Patient 7	Patient 8	Patient 9
irritability, sleep disorder, fever, abdominal pain, polydipsia, itching, loss of appetite	abdominal pain, fever, exanthema, itching, appetite loss	sleep disorder, subfebrility, exanthema, feeding difficulty	agressivity, abdominal pain, enuresis, itching, exanthema	irritability, sleep disorder, abdominal pain, sweating, exanthema, itching, loss of appetite	irritability, sleep disorder, limb pain	irritability, sleep disorder, enuresis, sweating, exanthema, sialorrhea, itching	irritability, exanthema, loss of appetite	sleep disorder, abdominal pain, polydipsia, sweating

The first symptoms associated with the neurological disease appear after the prodromal phase.

**Figure 1:**
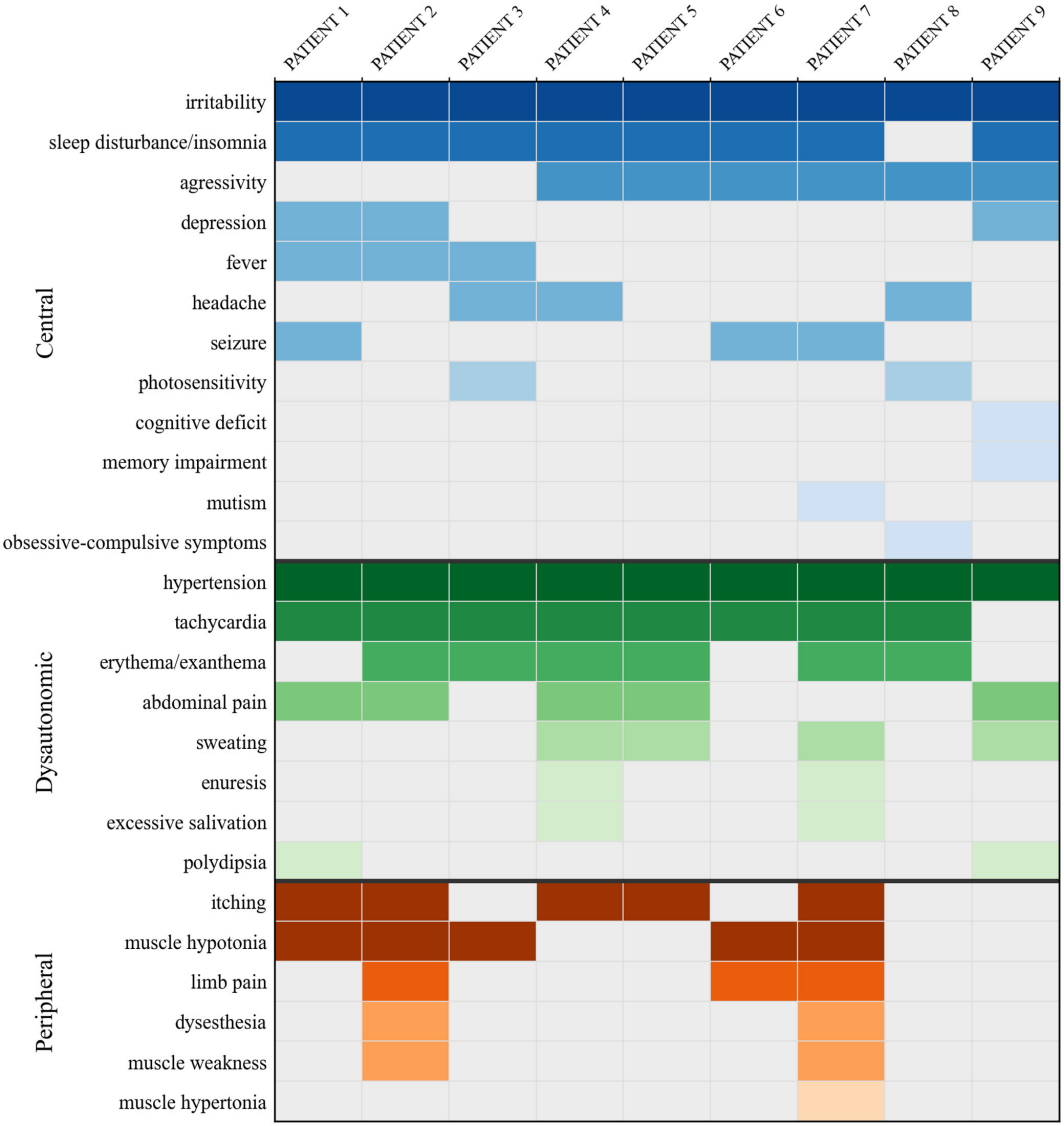
Main central, dysautonomic and peripheral symptoms.

**Figure 2:**
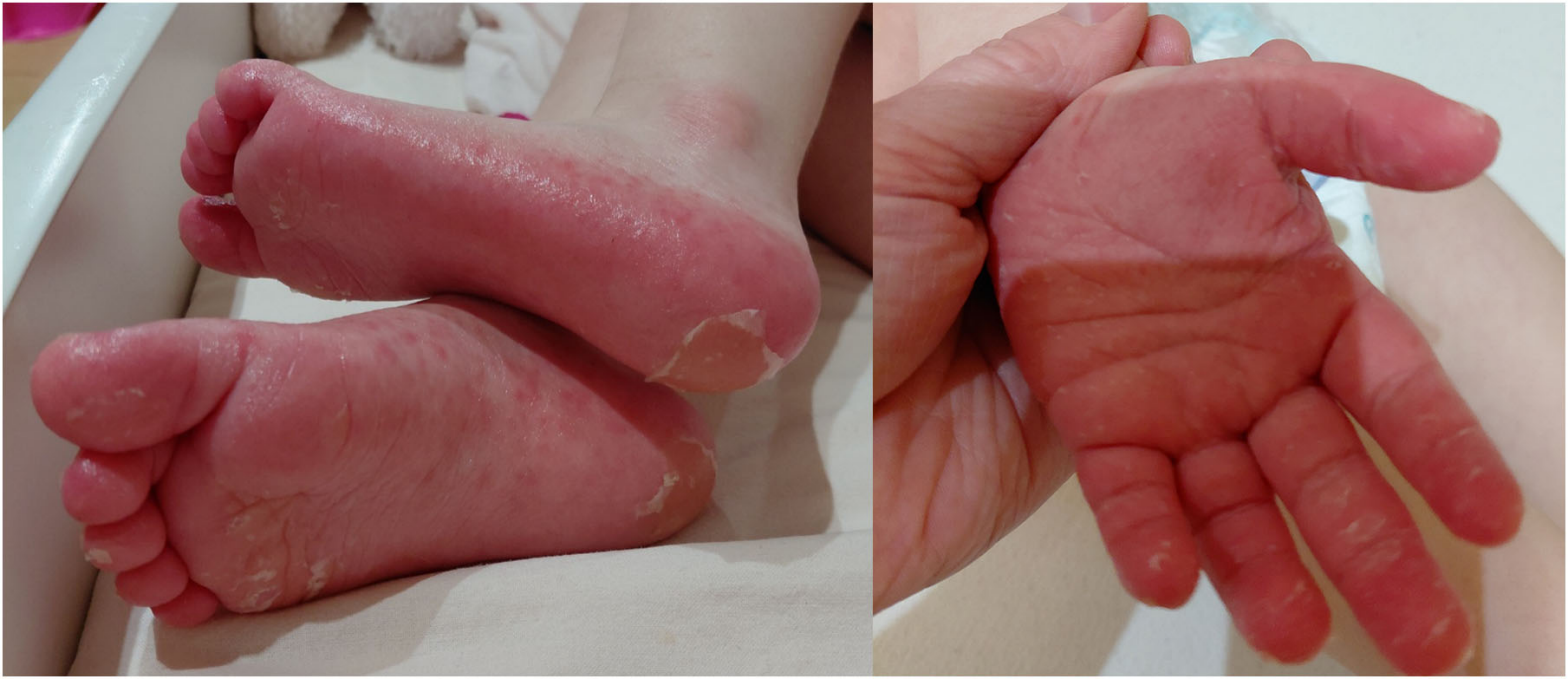
Palmoplantar erythematous skin lesions of patient 4. (The photographs are published with the parents’ written informed consent).

The most prevalent central nervous system manifestation was sleep disorder (8/9) (**Figure 1**). Among the neuropsychiatric symptoms, irritability (9/9) and aggressiveness (6/9) were the most common (**Figure 1**). Headache, fever, and seizures each occurred in 3/9 patients, whereas memory impairment and cognitive dysfunction were each present in only one patient (**Figure 1**). Neurological examination revealed abnormalities in 7/9 patients: absent reflexes (2/9), pyramidal signs (3/9), ataxia (3/9), somnolence (6/9), and hypotonia (4/9) (**Table 1**). The most common dysautonomic manifestations were hypertension (9/9), tachycardia (8/9), exanthema and/or erythema (6/9) and abdominal pain (5/9) (**Figures 1**, **2**). The most frequent peripheral nervous system symptoms included pruritus (5/9) (**Figure 1**). Additional symptoms included drowsiness and loss of appetite in 6/9 patients each, weight loss in 5/9, feeding difficulties in 3/9 and uncontrollable crying in 2/9 patients.

Four patients required intensive care. Patient 1 was admitted for status epilepticus, severe hypertension, and treatment-resistant hyponatremia. The hyponatremia did not improve with 3% NaCl infusion, but resolved after fludrocortisone administration. Patient 6 and 8 were admitted because of severe hypertension, and Patient 9 was admitted for management of PEX.

### 3.3 Laboratory findings

The mean delay to definitive diagnosis was 7.7 ± 4.2 weeks. All patients were positive for serum CASPR2 antibodies; 2/9 patients also showed CSF positivity. Serum anti-LGI1 antibodies were detected in 5/9 patients, with CSF positivity in 1/9 cases (**Table 1**). No CSF pleocytosis was observed, and elevated CSF protein levels were detected only in Patient 1 (890 mg/L). Intrathecal immunoglobulin synthesis was not detected in any patient.

Leukocytosis (>10 × 10^9^/L) after admission was present in 7/9 patients; CRP was normal in all patients (**Table 1**). Hyponatremia was detected in two patients (lowest serum sodium levels: 108 mmol/L in Patient 1 and 128 mmol/L in Patient 7, respectively). In Patient 3, renin and angiotensin levels were elevated; however, after further evaluation and nephrology consultation, these were considered part of an acute-phase response. Tumor markers showed mildly elevated NSE levels in Patients 2 and 3, and slight AFP elevation in Patient 3. These findings were not considered suggestive of malignancy by the oncologists. Paraneoplastic, and other autoimmune antibodies, immunoglobulin level assessment and complement studies revealed no clinically relevant abnormalities. In Patient 1 with severe treatment-resistant hypertension, catecholamine levels were mildly elevated.

### 3.4 Imaging and electrophysiology findings, differential diagnostic workup

Brain MRI findings were normal in 6/9 patients. Detected abnormalities included focal cortical/subcortical T2/FLAIR hyperintensities (Patients 3 and 6) and hippocampal-amygdala hyperintensity in Patient 7 (**Table 1**). No malignancy was identified on chest MRI or CT, abdominal MRI/CT or echocardiography; however mild left ventricular hypertrophy was noted in Patients 4 and 8. EEG abnormalities were observed in 4/9 patients, including generalized slowing (3/9) or right parieto-occipital delta bursts (Patient 6) (**Table 1**). ENG was performed in 3/9 patients and revealed possible sensory nerve demyelination in Patient 5. EMG was performed in 2/9 patients and suggested a neurogenic lesion in Patient 2. Comprehensive ophthalmologic, otorhinolaryngological, nephrological and gastroenterological evaluations were conducted and alternative etiologies were excluded.

### 3.5 Therapy

Immunotherapy was initiated after a mean delay of 8.8 ± 4.4 weeks from symptom onset. Five patients received combined IVIG and high-dose methylprednisolone (HDMP), one patient received IVIG alone, one received HDMP alone and one improved without immunotherapy. One patient initially received HDMP, but due to an insufficient clinical response, treatment was escalated to PEX (Patient 9). This patient subsequently started maintenance immunotherapy with azathioprine (**Table 1**). Oral corticosteroid maintenance therapy was administered in 7/9 patients for 9.5 ± 12.1 months. Antihypertensive treatment was started in 7/9 patients for 3.4 ± 1.7 months and required combination therapy in two cases. Due to relapse, Patient 3 also started maintenance immunotherapy (azathioprine) in addition to repeated HDMP treatment (**Table 1**).

### 3.6 Follow-up

Over a mean follow-up period of 3.0 ± 2.6 years, all patients survived. At onset, mRS scores ranged from 2 to 5 (3.2 ± 1.3); at discharge, 0 to 2 (0.9 ± 0.9). Two patients had persistent deficits including borderline cognitive performance and attention deficit in Patient 1, and muscle weakness in Patient 2. Patient 3 experienced relapse six months after prolonged steroid tapering, predominantly with peripheral nervous system symptoms.

## 4 Discussion

In this study, we present a case series of nine patients diagnosed with CASPR2 encephalitis, detailing their clinical presentation, laboratory and neuroimaging findings, electrophysiological results, therapeutic interventions and clinical outcomes. The cohort exhibited a slight male predominance, consistent with previous reports (2).

As both the present data and previous reports indicate, the diagnostic delay in CASPR2 encephalitis remains considerable (8–10). This underscores the importance of recognizing the broad spectrum of presenting symptoms and highlights the need to increase awareness of the clinical heterogeneity of this disease. Diagnosing pediatric autoimmune encephalitis is particularly challenging due to its heterogeneous, non characteristic, age-dependent and difficult-to-objectify clinical manifestations, as well as its subacute onset as observed in this cohort (**Figure 1**; **Table 2**) (7). Owing to these initially non-characteristic symptoms, some patients who were not referred to a pediatric neurologist may have remained undiagnosed. Several symptoms observed during the disease course can be particularly distressing for patients and their families, such as psychiatric manifestations and pain, whereas others, including severe hypertension, may pose direct life-threatening risks to the patient (2, 11). Certain manifestations, such as sleep disturbance, often require a high degree of clinical suspicion, as patients, particularly younger children, or their parents may not report them spontaneously (5). In other cases, the initial symptoms, which are often peripheral or dysautonomic in nature, may mimic other systemic disorders, for example rheumatologic or infectious diseases, thereby contributing to diagnostic delay (2, 7, 11, 12). Furthermore, several manifestations commonly observed in adults, such as hallucinations or other neuropsychiatric symptoms, are difficult to assess in young children (13).

In this case series, most patients exhibited central, peripheral, and dysautonomic symptoms. Among the core features observed in adults, autonomic dysfunction, insomnia and weight loss were also present in this pediatric cohort (5). Psychiatric symptoms and sleep disorders were highly prevalent, further supporting their prominent role in pediatric CASPR2 encephalitis (2, 14). Refractory hypertension was highly prevalent in the cohort and frequently mimicked other systemic diseases, as previously reported (11). Interestingly, many patients had skin manifestations, including exanthema and erythema, mostly in palmoplantar localization (**Figure 2**) (7). These lesions may result from vasomotor dysfunction driven by autonomic instability (11, 15, 16). Importantly, these dermatological manifestations should raise clinical suspicion and be differentiated from other pediatric diseases, such as enterovirus, parvovirus B19 infection and Kawasaki disease, particularly when accompanied by treatment-resistant hypertension and central nervous system manifestations (11, 17). Seizures, cognitive impairment, and cerebellar dysfunction were observed in only a minority of patients; however, these symptoms are considered core clinical features of the disease in adults (3, 5). Movement disorders were also less frequent in this cohort than in a previous pediatric study reporting a prevalence of 60% (2). In adults, amnesia and hallucinations are also common during the disease course, however, these manifestations were not observed either in this cohort or in previously published pediatric series (11, 18). Other symptoms, such as neuromyotonia accompanied by cramps and fasciculations may not be present in prepubertal children (11).

Based on the presented patients, the most common symptoms of pediatric CASPR2 encephalitis differ not only from those observed in adults but also from other forms of autoimmune encephalitis, such as NMDA encephalitis, which typically presents with language disturbances, psychiatric symptoms, and dyskinesia or dystonia (2). Nevertheless, some patients with CASPR2 encephalitis appeared to follow a clinical course with phases reminiscent of NMDA encephalitis (19).

In the present cohort, all patients had positive serum CASPR2 antibodies; only a minority demonstrated CSF positivity, consistent with previous findings (2, 20). It should be noted, that Patient 6 and Patient 9 had low serum CASPR2 antibody titers (1:40 and 1:20, respectively), which may raise the possibility of false-positive results (2). Both of the aforementioned patients exhibited clinical features consistent with CASPR2 encephalitis. Their improvement following immunotherapy, together with the exclusion of alternative diagnoses, supported an immune-mediated etiology, as similarly reported in previous case series (1, 2, 4). Furthermore, unlike NMDA antibodies, which are more frequently detected in the CSF, CASPR2 antibodies are more commonly identified in serum (1, 2, 6, 14, 20, 21). These findings highlight the ongoing challenge in interpreting the diagnostic significance of low serum CASPR2 antibody titers (2, 14).

We observed a high rate of co-occurrence of CASPR2 and LGI1 antibodies (5/9 patients, 56%) (1, 2, 22). LGI1 and CASPR2 are the two principal antigens within the voltage-gated potassium channel complex (23). In adult patients with LGI1 encephalitis, faciobrachial dystonic seizures and hyponatremia are the most characteristic clinical features (5, 23). However, CASPR2/LGI1 antibody double-positive patients rarely present with faciobrachial dystonic seizures, in accordance with the current results (22, 24). Hyponatremia has been reported in up to 60–80% of patients (23–25), potentially explained by the ability of LGI1 antibodies to bind to antidiuretic hormone-secreting neurons (22). Therefore, in Patient 7, LGI1 co-positivity may explain the occurrence of hyponatremia. However, in Patient 1, repeated autoimmune encephalitis antibody testing was performed on multiple occasions, and none of the analyses demonstrated LGI1 antibody positivity. Although hyponatremia in CASPR2 encephalitis without concomitant LGI1 antibody positivity has been reported in the literature, such cases appear to be rare (22, 26). It should be also noted, that in three patients (Patient 2-4), serum LGI1 antibody levels were only slightly elevated and could not be detected during follow-up testing, whereas CASPR2 antibodies remained detectable during the symptomatic phase of the disease.

Other laboratory findings are usually nonspecific for CASPR2 encephalitis (2). In the cohort, CSF analysis revealed elevated protein levels in only one patient, in accordance with the literature reporting that standard CSF laboratory tests may yield unremarkable results in 75% of cases (1, 5, 27). Patient 1, who presented with treatment-resistant hypertension, also demonstrated elevated catecholamine levels, a phenomenon previously described as evidence of complex autonomic and endocrine dysregulation in this disease (11).

Most patients had normal or nonspecific MRI findings, in accordance with previous reports (2, 4, 14, 27). Brain abnormalities may involve the cerebral cortex, thalamus, caudate nucleus, white matter, hippocampus, globus pallidus and corpus callosum (2, 4, 14, 27). One possible explanation for this phenomenon is that MRI findings may be normal at disease onset but may become apparent during follow-up (4).

In adults, malignancies, particularly thymoma in patients with Morvan syndrome, may underlie CASPR2 encephalitis; however, no tumors were identified in this cohort, consistent with prior pediatric reports (4, 5, 7, 14).

The conventional treatment of CASPR2 encephalitis does not differ from other autoimmune encephalitides (2). Nearly all patients received first-line immunotherapy with favorable clinical responses, consistent with previous studies (2, 5, 7). Generally, patients have poorer outcome without immunotherapy, however, complete recovery without immunotherapy may occur in some cases, as observed in Patient 8 (28). In a large cohort of patients, favorable outcomes were reported (mRS ≤2) in 73% of patients, in accordance with the present findings (3). Nevertheless, it is important to emphasize that the disease generally has a favorable prognosis when diagnosed early, as supported by the presented results.

CASPR2 encephalitis carries a risk of recurrence in adults (up to 25%), although this is rarely reported in children (1–4, 6). Despite a prolonged corticosteroid tapering, Patient 3 experienced a relapse six months after steroid withdrawal. Interestingly, the clinical presentation differed from that of the initial episode and was limited to peripheral symptoms, making clinical suspicion particularly challenging. Similar phenotypic variations during relapse have also been described in adults (5).

## 5 Conclusions

CASPR2 encephalitis in children may be underrecognized because of its heterogeneous clinical presentation and the difficulty of assessing certain symptoms in younger patients. The most common symptoms often differ from those observed in adults and in other types of autoimmune encephalitis. Certain symptom constellations may facilitate earlier recognition of CASPR2 encephalitis, including sleep disturbances, behavioral changes, otherwise unexplained hypertension accompanied by tachycardia, weight loss and the presence of palmoplantar exanthema or erythema. Recognizing these features may help clinicians consider CASPR2 encephalitis earlier, especially in cases with otherwise non-characteristic presentations.

Although no standardized treatment protocol currently exists, favorable outcomes can be achieved with immunotherapy. This series underscores the broad clinical spectrum of CASPR2 encephalitis and highlights the importance of a detailed clinical history and carefully evaluating of patient-reported symptoms. Early diagnosis is crucial for achieving favorable clinical outcomes. Therefore, prospective, large-scale studies are needed to further characterize the clinical spectrum, imaging findings and laboratory findings, treatment responses, and prognostic factors in pediatric CASPR2 encephalitis.

### Limitations

First, the small sample size precluded meaningful statistical comparisons and limits the generalizability of the findings. In addition, the retrospective multicenter design carries an inherent risk of incomplete or non-uniform data collection across participating centers. Because all cases originated from Hungarian centers, the cohort may also be affected by ethnic or referral-pattern bias. Furthermore, the variability in follow-up duration restricted the assessment of long-term clinical outcomes. Finally, the clinical significance of low-titer serum CASPR2 antibody positivity observed in Patients 6 and 9 remains uncertain, and false-positive test results cannot be excluded.

## Data Availability

The original contributions presented in the study are included in the article/supplementary material. Further inquiries can be directed to the corresponding author.
